# *Strongyloides stercoralis* genotyping in a human population in southwestern Iran

**DOI:** 10.1186/s13071-023-06103-6

**Published:** 2024-01-16

**Authors:** Molouk Beiromvand, Alireza Ashiri, Veroni de Ree, Dorothee Harbecke, Christian Rödelsperger, Adrian Streit, Abdollah Rafiei

**Affiliations:** 1 Infectious and Tropical Diseases Research Center, Health Research Institute, Ahvaz Jundishapur University of Medical Sciences, Ahvaz, Iran; 2 Department of Parasitology, School of Medicine, Ahvaz Jundishapur University of Medical Sciences, Ahvaz, Iran; 3 Department of Integrative Evolutionary Biology, Max Planck Institute for Biology Tübingen, Tübingen, Baden‑Württemberg Germany

**Keywords:** Strongyloidiasis, *Strongyloides stercoralis*, Iran, Molecular taxonomy, Whole-genome sequencing

## Abstract

**Background:**

Strongyloidiasis is a neglected tropical disease (NTD) that is caused mainly by *Strongyloides stercoralis*, with an estimated 600 million people infected worldwide, and in fewer cases by *Strongyloides fuelleborni fuelleborni* and *Strongyloides fuelleborni kellyi*. A number of studies have been conducted on the genetic diversity of *S. stercoralis* in East and Southeast Asia; however, there is very limited corresponding information from West Asian countries, including Iran.

**Methods:**

For *Strongyloides* worms collected from patients in southwestern Iran, the hypervariable regions I (HVR-I) and IV (HVR-IV) of the nuclear 18S ribosomal DNA (rDNA) locus (*SSU*) and a fragment of the subunit 1 mitochondrial cytochrome *c* oxidase gene (*cox-1*) were sequenced. For a subset of the worms, whole-genome sequencing data were generated.

**Results:**

The *cox-1* sequences of 136 worms isolated from 23 patients indicated that all isolates were *S. stercoralis.* Among the *cox-1* sequences, 33 polymorphic sites and 13 haplotypes were found. The phylogenetic analysis demonstrated that some sequences clustered fairly closely with sequences from humans and dogs from other parts of the world, while others formed a separate, Iran-specific group. Among 64 *S. stercoralis* analyzed, we found three of the previously described *SSU* HVR-I haplotypes, with haplotype II being the most frequent haplotype. In contrast to Southeast Asia, where *S. stercoralis* heterozygous for different haplotypes at the HVR-I locus are rare, we found 20 worms to be heterozygous for two different HVR-I haplotypes, 18 of which fell into the Iran-specific *cox-1* cluster. *SSU*-heterozygous worms also showed elevated heterozygosity at the whole-genome level.

**Conclusions:**

We conclude that the *S. stercoralis* population from the Khuzestan province shares much of the genetic diversity with the population in Southeast Asia, but there is an indication of additional genetic input. There appears to be some population structure with different subpopulations, which however do interbreed at least occasionally.

**Graphical Abstract:**

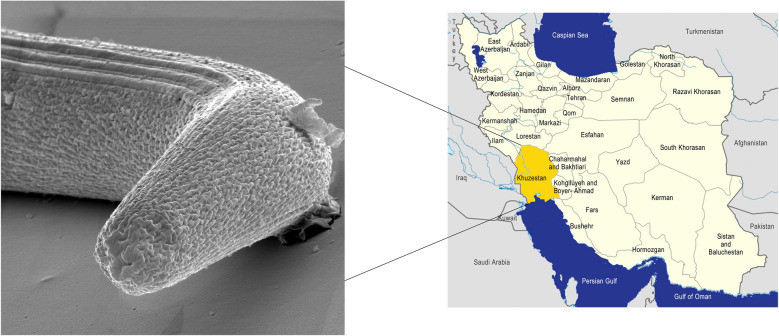

**Supplementary Information:**

The online version contains supplementary material available at 10.1186/s13071-023-06103-6.

## Background

As one of the most common neglected tropical diseases (NTDs) [1], strongyloidiasis is an intestinal infection affecting about 600 million individuals, predominantly in tropical and subtropical areas [2, 3]. The disease is caused by *Strongyloides stercoralis* and, to a much lesser extent, *Strongyloides fuelleborni fuelleborni* and *Strongyloides fuelleborni kellyi*. In addition to humans, *S. stercoralis* can infect certain animals, including non-human primates (NHPs), cats, and dogs [2, 4, 5]. *Strongyloides stercoralis* is a soil-transmitted helminth with a unique life cycle alternating between free-living and parasitic cycles [6]. Moreover, its internal autoinfection cycle lets it persist in the host's body for decades [6]. Most patients with *S. stercoralis* are asymptomatic [7]. However, several gastrointestinal manifestations, including abdominal pain, diarrhea, and constipation, have been observed in acute and chronic infections [8]. Also, it has been shown that hyperinfection and disseminated strongyloidiasis, which may result from a loss of control of the autoinfection cycle predominantly in immunocompromised patients, is fatal in 85–100% of cases [9]. A lack of early diagnosis and effective treatment can significantly increase *S. stercoralis*-related mortality and morbidity [10].

Strongyloidiasis is usually diagnosed using traditional and routine laboratory methods, such as the Baermann technique or agar plate culture (APC), followed by light microscopy. However, these methods are somewhat limited because of their low sensitivity in mild and chronic infections, and their time-consuming culture process [11]. Moreover, detection of *S. stercoralis* larvae is not always possible, especially in chronic strongyloidiasis, due to intermittent and low egg-laying rates [6]. Therefore, advanced techniques such as molecular and serological methods are sometimes used to overcome the limitations of traditional parasitological methods. However, even these techniques may not have enough sensitivity in acute cases, and they tend to have specificity issues because false-positive results may arise due to cross-reactions with other nematodes [5].

Several studies have investigated the diagnosis, treatment, and epidemiology of *S. stercoralis* in different areas [12–14], and a number of studies distributed over several decades have investigated the zoonotic properties of this parasite, yielding controversial results [15]. However, only a few of these studies included genetic/genomic investigations of this nematode, and the studies that did were geographically heavily biased towards East Asia and Australia [14, 16–19]. Therefore, a comprehensive genomic analysis of this nematode is needed, including its genotypes in various hosts from different areas [2].

Ramachandran et al. [20] were the first to investigate *Strongyloides* using a molecular genetics approach by analyzing a part of its genome from the gene of 18S ribosomal RNA (rRNA) to the gene of 28S rRNA using the polymerase chain reaction–restriction fragment length polymorphism (PCR–RFLP) method [20]. Hasegawa et al. [21] subsequently investigated the 18S ribosomal DNA (rDNA) of this nematode to find the sequences that could be used for species–specific diagnosis. This study introduced four hypervariable regions (HVRs), HVR-I to HVR-IV, in the 18S rRNA gene as diagnostic markers for genotyping *Strongyloides* spp. [21]. Since different isolates of *S. stercoralis* from humans, dogs, and chimpanzees tended to show little or no variation in these HVRs, Hasegawa et al. [21] proposed a 722-base-pair (bp) fragment of the mitochondrial cytochrome *c* oxidase subunit 1 gene (*cox-1*) as a suitable genotyping marker for detecting intraspecific variation [21]. Several recent studies have investigated the HVR-I, HVR-IV, *cox-1*, and whole-genome sequences of *Strongyloides* spp. isolated from Southeast Asia, Japan, China, and Australia to identify intraspecific variations [14, 16, 17, 22].

Iran is an endemic area for *S. stercoralis* [23]. However, molecular genetic/genomic information about *S. stercoralis* in Iran is extremely limited. While a limited number of *cox-1* and partial 18S sequences derived from *S. stercoralis* from Iran are available in GenBank (search for “cytochrome OR 18S AND *Strongyloides* AND Iran” on the GenBank website [https://www.ncbi.nlm.nih.gov/nuccore] on October 14, 2022, and [24]), to our knowledge, no full-genome information about *S. stercoralis* from Iran has been reported. Therefore, the present study analyzed the HVR-I and HVR-IV of the nuclear 18S rDNA locus (*SSU*), *cox-1*, and the whole-genome sequences of the isolates of *S. stercoralis* collected from the human population of Khuzestan province, located in southwestern Iran.

## Methods

### Study area and sample collection

Khuzestan province, which is located in southwestern Iran near the border of Iran and Iraq, has hot and sometimes humid summers, especially in the south, and cold and dry winters. The province has an area of 63,238 km^2^ and a population of over 4.7 million individuals [25].

**Fig. 1:**
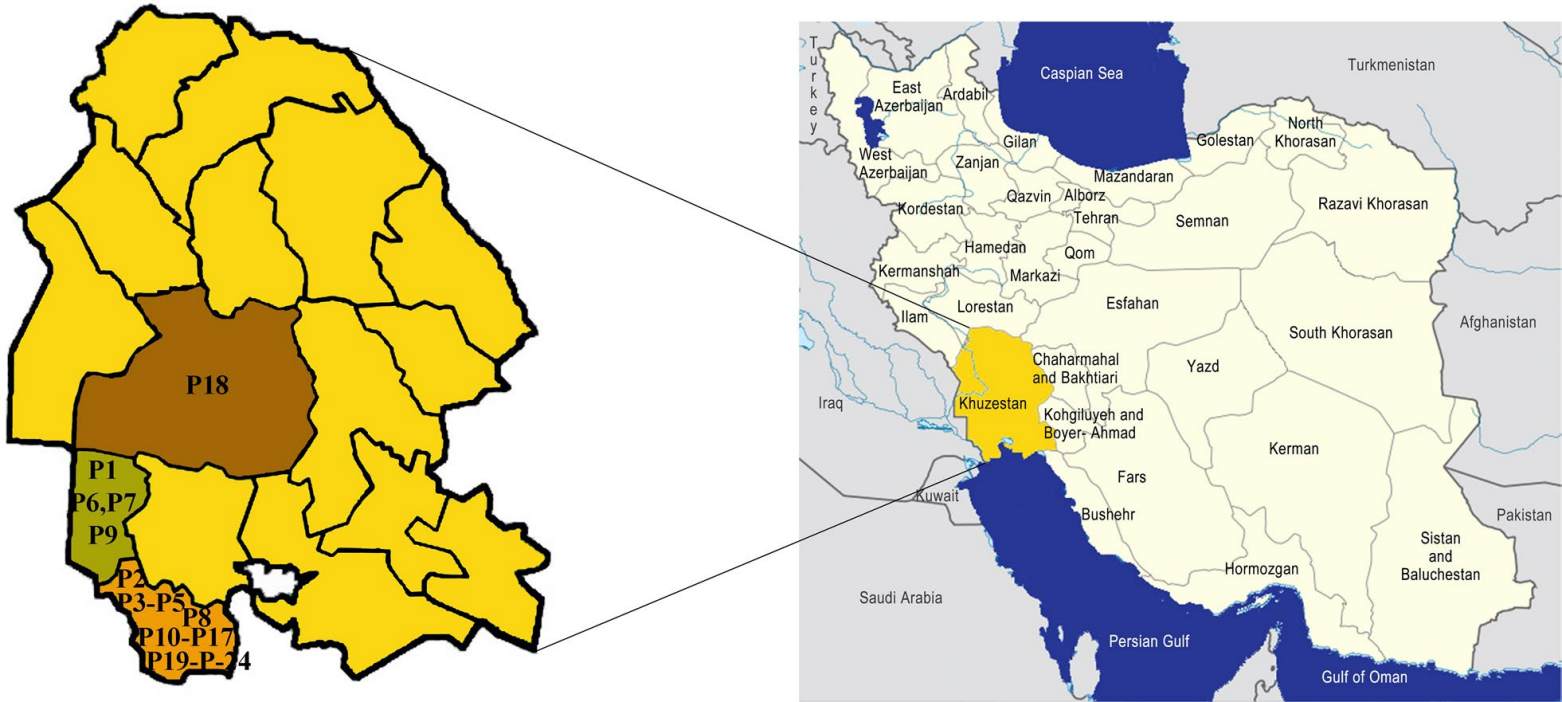
Map of the sampling area. The origin of the patients (P[number]) is indicated

Twenty-three patients who had been referred to hospitals in the southern counties of Khuzestan province, including Abadan, Khorramshahr, and Ahvaz, for different reasons but found to be infected with *Strongyloides* spp. were enrolled in the present study (Fig. 1). The patients with strongyloidiasis were first diagnosed using direct smear examination with a light microscope under ×100 and ×400 magnification. Then, their infections were confirmed by observing the morphological characteristics of *S. stercoralis* cultured using the APC method [26]. After that, the infective larvae and adult worms were transferred to 1.5-ml tubes containing 80% ethanol and stored at −80 °C. The samples were then sent to the Department of Integrative Evolutionary Biology, Max Planck Institute for Biology, Tübingen, Germany, for molecular analyses.

### Lysis of *S. stercoralis* adult worms and larvae

The samples containing *S. stercoralis* worms and larvae fixed in ethanol were transferred to a watch glass and rinsed at least three times with tap water. Afterward, each adult worm or larva was picked and transferred to a PCR tube containing 10 µl of PCR water. The samples were then frozen in liquid nitrogen and thawed at room temperature; this process was carried out three times. Afterward, 10 µl of 2× lysis buffer (20 mM Tris–HCl with a pH of 8.3, 100 mM KCl, 5 mM MgCl_2_, 0.9% NP-40, 0.9% Tween 20, and 240 μg/ml proteinase K) was added to each tube. The tubes were then incubated at 65 °C for 2 h. The resulting lysate was stored at −20 °C until further examination.

### PCR amplification of *SSU* and *cox-1*

Three molecular markers (18S HVR-I, 18S HVR-IV, and *cox-1*) were amplified using the primers described by Zhou et. al. [19] (Table 1). A fragment of the nuclear *SSU* gene of *S. stercoralis*, 18S rRNA (HVR-I), with a length of about 862-bp was amplified using 10 μl of Thermo Scientific DreamTaq Green PCR Master Mix (2×), 0.25 μM of each primer, 8 μl of nuclease-free water, and 1 μl of template DNA in a final volume of 20 μl. The PCR program included an initial denaturation at 95 °C for 1 min, followed by 35 cycles of denaturation at 95 °C for 20 s, annealing at 52 °C for 15 s, extension at 72 °C for 90 s, and final extension at 72 °C for 5 min [22].

**Table 1:** Sequences, annealing temperatures, and product size of PCR primers used in the current study^a^

Target gene	Primers	Nucleotide sequences (5′–3′)	Annealing temp (°C)	Product length
18S rRNA (HVR-I)	Forward (RH5401)	AAACATGAAACCGCGGAAAG	52	862 bp
	Reverse (RH5402)	CATTCTTGGCAAATGCTTTCG		
	Sequencing (RH5403)	AGCTGGAATTACCGCGGCTG		
18S rRNA (HVR-IV)	Forward (18SP4F)	GCGAAAGCATTTGCCAA	57	712 bp
	Reverse (18SPCR)	ACGGCCGGTGTGTAC		
	Sequencing (ZS6269)	GTGGTGCATGGCCGTTC		
*cox-1*	Forward (ZS6985)	GGTGGTTTTGGTAATTGAATG	47	837 bp
	Reverse (ZS6986)	ACCAGTYAAACCACCAATAGTAA		
	Sequencing (ZS6990)	GGTTGATAAACTATAACAGTACC		

^a^All primers were taken from Zhou et. al. [19]

In addition, a 712-bp fragment of the nuclear *SSU* gene, the 18S rRNA (HVR-IV), was amplified using 10 μl of Thermo Scientific DreamTaq Green PCR Master Mix (2×), 0.2 μM of each primer, 7.2 μl of nuclease-free water, and 2 μl of DNA template in a final volume of 20 μl. The program consisted of one cycle at 94 °C for 2 min, followed by 35 cycles of denaturation at 94 °C for 30 s, annealing at 57 °C for 15 s, extension at 72 °C 90 s, and final extension at 72 °C for 10 min. Also, an 837-bp fragment of the mitochondrial *cox-1* gene was amplified using 10 μl of Thermo Scientific DreamTaq Green PCR Master Mix (2×), 0.2 μM of each primer, and 1 μl of the DNA template adjusted to 20 μl with nuclease-free water. The PCR program was as follows: initial denaturation at 95 °C for 30 s, followed by 35 cycles of denaturation at 95 °C for 20 s, annealing at 47 °C for 15 s, extension at 68 °C for 90 s, and final extension at 68 °C for 5 min.

### Sequencing

**Fig. 2:**
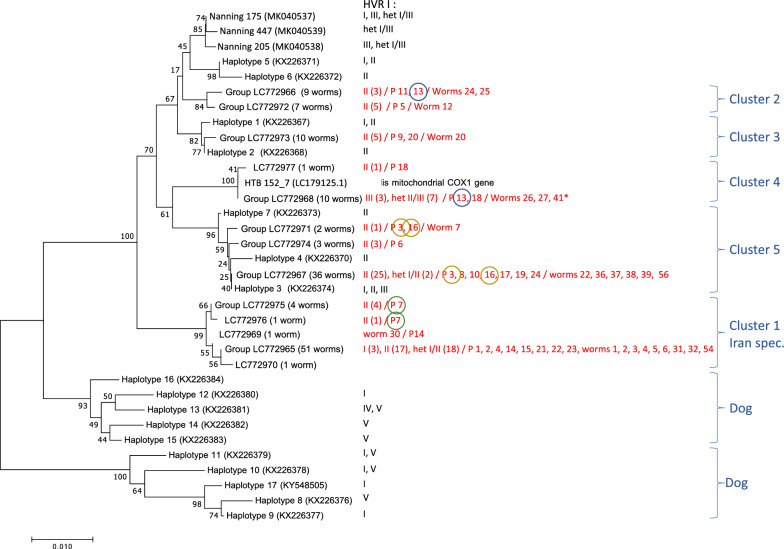
Neighbor-joining tree based on 552 bp of the mitochondrial *cox-1* gene. In total, 136 *Strongyloides stercoralis* worms from Iran are represented. For comparison, published sequences are included. Each haplotype is included in the tree only once, except for the one case where a haplotype (LC772967) was identical to a previously reported haplotype (KX226374 [14]). To the right, the nuclear *SSU* HVR-I haplotypes (nomenclature according to [14, 32] that were present among the bearers of the particular *cox-1* haplotype are indicated, if known. Values in parentheses indicate the number of worms with this haplotype (note that the *SSU* HVR-I haplotype is not known for all the worms for which the *cox-1* sequence was determined). Results from this study are in red. For these also, the patients with worms with this haplotype and the worms selected for whole-genome sequencing are indicated. Patients with worms of more than one *cox-1* sequence are circled. The blue brackets show clusters. The two dog clusters are from [14]. Scale bar denotes 0.01 changes per nucleotide site. *Note that *SSU* HVR-I haplotypes II and III differ by only one nucleotide (TTT in haplotype II and TAT in haplotype III). Distinguishing homozygous III and heterozygous II/III is therefore not obvious. All three whole-genome-sequenced worms of this group turned out to be heterozygous, although only one of them had been scored as heterozygous based on the HVR-I sequencing alone. Labels: Samples starting with “Nanning” are from [19], samples starting with “Haplotype” are from [14], HTB 152_7 is from [17], samples starting with “Group” are sequences from this study and were found in multiple worms (the number of worms with the particular haplotype is indicated in parentheses), and plain accession numbers are from this study and were found in only one worm

For sequencing, 1 μl PCR product was mixed with 1 μl of sequencing primer (Table 1) and 8 μl of nuclease-free water and submitted for Sanger sequencing to Genewiz, Leipzig, Germany. The Sanger sequencing data were analyzed using SeqMan Pro version 17.3 (Lasergene package; DNASTAR, Inc., Madison, WI, USA) and were compared with the sequences previously deposited in GenBank at the National Center for Biotechnology Information (NCBI). The accession numbers and the corresponding references are given in Fig. 2. The number of polymorphic sites, average number of pairwise nucleotide differences (K), nucleotide diversity (Pi), and haplotype (gene) diversity (Hd) were calculated using DnaSP version 6. The trees were constructed using MEGA 7 with the neighbor-joining method, and evaluated with 1000 bootstrap repetitions. The evolutionary distances were computed using the Kimura 2-parameter method. The use of the different models resulted in essentially the same tree topology.

### Whole-genome sequencing

The protocol for library construction for Illumina whole-genome sequencing was based on a previously described protocol [22]. This protocol was modified according to suggestions by Kohta Yoshida. The modified protocol is described below (for buffer compositions see [22]):

### DNA clean-up

A total of 10 μl of single worm lysate was added to a mixture of 10 μl of nuclease-free water, 16 μl of PEG8000/NaCl, and 4 µl of Sera-Mag beads in PEG8000/NaCl. The tube's contents were mixed and left at room temperature for 10 min. The tubes were then placed on a magnetic rack for 5 min at room temperature, the supernatant was discarded, and the remaining content (beads) was washed twice with 200 μl of 80% ethanol while the tube was on the magnet. Afterward, the beads were left to dry for a few minutes until all the remaining ethanol had evaporated. The tubes were removed from the magnetic rack, and 9 μl of Tris–HCl (10 mM, pH = 8.0) was added to the beads. Following complete mixing through pipetting, the tube was incubated for 10 min at room temperature. The samples were then placed on a magnetic stand for 5 min. Finally, 7 μl of the supernatant was transferred to a new tube without disturbing the beads.

### DNA tagmentation

The DNA tagmentation was performed as follows: To the 7 μl supernatant, which included the DNA, 5 μl of the Tn5 reaction solution (consisting of 2 μl of water, 2 μl of 5× [tris(hydroxymethyl)methylamino]propanesulfonic acid–dimethylformamide [TAPS-DMF] buffer, and 1 μl of 25× diluted [in glycerol/dialysis buffer] Tn5 [taken from the Nextera DNA Library Prep Kit, Cat. No. FC-121-1030]) was added and mixed by pipetting. The resulting mixture was incubated twice for 7 min at 55 °C in a PCR machine with a lid temperature of 75 °C with mixing by finger tapping between the two incubation steps.

### PCR amplification and adapter extension

To the mixture from the previous step (12 µl), 10 μl of 5× Q5 buffer (High-Fidelity DNA Polymerase, New England Biolabs), 2 μl of deoxynucleotide triphosphates (dNTPs) (10 mM each), 2 μl of i5 barcoded Nextera primer (5 μM), 1 μl of i7 barcoded Nextera primer (5 μM), 0.5 μl of Q5 Hi-Fi polymerase, and 39.5 μl of water were added and thoroughly mixed. Afterward, the mixture underwent the following steps in a thermal cycler: one cycle at 72 °C for 4 min, one cycle at 98 °C for 30 s, 14 cycles of denaturation at 98 °C for 15 s, annealing at 67 °C for 20 s, extension at 72 °C for 90 s, and cooling to 4 °C.

### Quality check and size selection

For checking the DNA quality and length, 18 samples which included 10 adult worms and eight larvae were randomly selected, and 5 μl of PCR reaction mix was evaluated by gel electrophoresis on a 1.5% agarose 1× tris-acetate-ethylenediaminetetraacetic acid (TAE) gel at 70 V for 5 min and 100 V for 25 min. Afterward, the DNA molecules between 600 and 300 bp were enriched using Sera-Mag beads following the published protocol [22].

### Quantification of concentration and size

The DNA concentration in all samples was assessed using the Qubit 2.0 Fluorometer (Thermo Fisher Scientific) and the fragment lengths were determined on an Agilent 2100 Bioanalyzer, following the manufacturers’ instructions.

### Pooling and concentration adjustment

Based on the DNA concentration determined above, 60 fmol of each library was pooled. The pooled volume was measured, and then 1.2× volume of Sera-Mag beads was added to the sample. The pooled sample was incubated for 10 min at room temperature and 5 min on a magnetic stand. After removing the supernatant, the beads were washed twice with 1 ml 80% ethanol while the tube was on the magnet, and the beads were left to dry for a few minutes. The tube was removed from the magnetic stand and the beads were resuspended in 18 μl Tris–HCl (10 mM, pH = 8.0) and incubated for 10 min at room temperature. The tube was then placed on a magnetic stand for 5 min. Finally, 16 μl of the supernatant was transferred to a new tube without disturbing the beads.

The concentration was measured using a Qubit (Thermo Fisher Scientific) and adjusted to 2.5 nM, and then submitted to the Max Planck Institute for Biology in-house sequencing facility for sequencing on an Illumina NextSeq 2000 instrument.

### Analysis of whole-genome sequencing data

Between 3.6 and 8.4 million read pairs were sequenced (2 × 150 bp) per sample, resulting in theoretical coverage of 20–60 times for the *S. stercoralis* genome (43 Mb). All reads were uploaded to the European Nucleotide Archive under the study accession PRJEB64686. The BWA-MEM program (version 0.7.17-r1188, default parameters) was used to align raw reads against the *S. stercoralis* genome from WormBase ParaSite (version WBPS11) [27–29]. Since in this genome assembly the *SSU* locus is not fully represented, the reads were also aligned to the 18S sequence (AF279916) in order to confirm the *SSU* HVR-I and HVR-IV haplotypes. The samtools program (version 1.18, view, sort, index, and rmdup commands) was run to generate binary alignment files and to remove duplicate reads, and initial variant calls were generated by combining the mpileup, bcftools view (version 0.1.17-dev), and vcfutils.pl varFilter (-D1000-w0 parameters) commands of the samtools program (version 0.1.18) [27]. Heterozygous sites were defined based on a positive consensus quality (FQ) value in the variant calling file. Variant positions with quality scores > 20 were pooled and genotyped in the alignment files of the current and previous studies using samtools [16, 19]. Variant positions that were genotyped in all samples were used for constructing a neighbor-joining tree with the help of the phangorn library in R [30] and for performing a principal component analysis (PCA) using the Eigensoft smartpca program (version 8.0.0) [31]. At this step, two samples with fewer than 30,000 genotyped variant positions were discarded. For heterozygosity analysis, we considered only samples where at least 85% of all variant positions could be genotyped with a quality score > 20.

## Results and discussion

Among the 23 examined *Strongyloides*-positive patients, ranging in age from 40 to 92 years and with a mean age of 70.2 years, 18 (78.3%) were male and the remaining five (21.7%) were female. The majority of patients (18/23) were from Abadan County and its dependent cities and rural regions, and the rest were residents of Khorramshahr (4/23) and Ahvaz counties (1/23), southwestern Iran. In total, 50 adult worms and 106 larvae were isolated from the patients and subjected to molecular analysis.

### *cox-1* Haplotypes

The PCR amplification and sequencing of *cox-1* was successful for 136 worms. The obtained sequences all had database entries derived from *S. stercoralis* as the best BLAST (Basic Local Alignment Search Tool) hits with very low e-values and, upon phylogenetic analysis, grouped with previously published *S. stercoralis* sequences (see Fig. 2, Additional file 1, for accession numbers and references), indicating that all isolates were *S. stercoralis.* Among the 136 *S. stercoralis cox-1* sequences, 33 polymorphic sites and 13 haplotypes were found (Table 2). The average number of pairwise nucleotide differences (552 bp) and nucleotide diversity among the 13 haplotypes were 11.949 and 0.02165, respectively (for pairwise comparisons see Additional file 2). To evaluate the phylogenetic relationships among the haplotypes from this study and with isolates from different geographical locations, a phylogenetic tree was constructed using the neighbor-joining method (Fig. 2, Additional file 1: Fig. S1). All sequences grouped with the sequences from the human and dog parasitic cluster of *S. stercoralis* [14, 17, 32]. Within this cluster, some sequences grouped fairly closely with sequences from other parts of the world, while others formed a separate Iran-specific group (Fig. 2). The closest database entry to this group is MK049075.1, which is derived from a worm isolated in the Khuzestan province. Since in this database entry the full fragment considered in this study is not available, this sequence is not included in Fig. 2. The most prevalent haplotype outside of the Iran-specific group (LC772967, 36 worms from seven different patients) was identical to haplotype 3 (KX226374) previously reported from humans and from dog-derived worms in Cambodia [14]).

**Table 2:** *Strongyloides stercoralis* with the different *cox-1* haplotypes from 23 patients

*cox-1* Haplotype^a^	Patient number, location^b^	*S. stercoralis* individuals with this sequence^c^	Number (%) of worms with this *cox-1* haplotype
LC772965 (cluster 1, Iran-spec.)	1 Khorramshahr 2 Abadan 4 Abadan **14** Abadan **15** Abadan 21 Abadan 22 Abadan 23 Abadan	A1-P1.adult (het)^d^ [Worm 1], B1-P1.adult (het)^d^ [Worm 2], C1-P1.adult (I or het), D1-P1.adult (het)^d^ [Worm 3], E1-P1.larva, F1-P1.larva (het), G1-P1. larva^d^ A2-P2.adult (het)^d^ [Worm 4], B2-P2.adult (het)^d^ [Worm 5], C2-P2.adult (het)^d^ [Worm 6], D2-P2.adult (het), E2-P2.larva (het), F2-P2.larva (het), G2-P2. larva (het) A4-P4.adult (II), B4-P4.larva (II), C4-P4.larva (II), D4-P4.larva (II), E4-P4.larva (II), F4-P4.larva (II) B12-P14.adult (II)^d^ [Worm 31], C12-P14.adult (II)^d^ [Worm 32], D12-P14.adult (II), E12-P14.larva (II), G12-P14.larva (II) B1-P15.larva (II), C1-P15.larva (het), D1-P15.larva, E1-P15.larva, F1-P15.larva, G1-P15.larva A7-P21.larva, B7-P21.larva, C7-P21.larva, D7-P21.larva (I), E7-P21.larva (I), F7-P21.larva, G7-P21.larva A8-P22.larva (het), B8-P22.larva (het), C8-P22.larva (het), D8-P22.larva (het), F8-P22. larva (het), G8-P22. larva (I) A9-P23.adult (II)^d^ [Worm 54], B9-P23.adult (II), C9-P23.adult (II), D9-P23.adult (II), E9-P23.larva^d^, F9-P23.larva, G9-P23. larva (II)	51 (37.5)
LC772966 (cluster 2)	11 Abadan **13** Abadan	A10-P11.adult (II)^d^ [Worm 24], B10-P11.adult (II)^d^ [Worm 25], C10-P11.larva, D10-P11.larva, G10-P11.larva, G11-P13.larva, F10-P11.larva (II) D11-P13.larva, E11-P13.larva	9 (6.62)
LC772967 (cluster 5)	**3** Abadan 8 Abadan 10 Abadan **16** Abadan 17 Abadan 19 Abadan 24 Abadan	B3-P3.adult (II), C3-P3.adult (II), D3-P3.adult (II), E3-P3.adult (II), F3-P3.larva (II), G3-P3.larva (II) B8-P8.larva, C8-P8.larva, D8-P8.larva A9-P10.adult (het)^d^ [Worm 22], B9-P10.larva (het) A2-P16.adult (II)^d^ [Worm 36], C2-P16.adult, D2-P16.adult (II)^d^ [Worm 37], E2-P16.larva (II), F2-P16.larva (II), G2-P16.larva A3-P17.adult (II)^d^ [Worm 38], B3-P17.adult (II)^d^ [Worm 39], C3-P17.adult (II), D3-P17.adult (II), E3-P17.larva (II), F3-P17.larva B5-P19.larva (II), C5-P19.larva (II), D5-P19.larva (II), E5-P19.larva (II), F5-P19.larva (II), G5-P19.larva (II) A10-P24.adult (II)^d^ [Worm 56], B10-P24.larva (II), C10-P24.larva, D10-P24.larva, E10-P24.larva, F10-P24.larva (II)^d^, G10-P24.larva (II)	36 (26.47)
LC772968 (cluster 4)	**13** Abadan **18** Ahvaz	A11-P13.adult (het II/III)^d^ [Worm 26], B11-P13.adult (het II/III)^d^ [Worm 27], C11-P13.larva (III), F11-P13.larva (het II/III) A4-P18.adult (III), B4-P18.adult (het II/III)^d^ [Worm 41], C4-P18.adult (het II/III), D4-P18.larva (het II/III), F4-P18.larva (III), G4-P18.larva (het II/III)	10 (7.35)
LC772969 (cluster 1, Iran-spec.)	**14** Abadan	A12-P14.adult (II)^d^ [Worm 30]	1 (0.74)
LC772970 (cluster 1, Iran-spec.)	**15** Abadan	A1-P15.larva^d^	1 (0.74)
LC772971 (cluster 5)	**3** Abadan **16** Abadan	A3-P3.adult (II)^d^ [Worm 7] B2-P16.adult	2 (1.47)
LC772972 (cluster 2)	5 Abadan	A5-P5.adult (II)^d^ [Worm 12], B5-P5.adult (II), C5-P5.adult (II), D5-P5.adult (II), E5-P5.larva, F5-P5.larva (II), G5-P5.larva	7 (5.14)
LC772973 (cluster 3)	9 Khorramshahr 20 Abadan	9-P9.adult (II)^d^ [Worm 20], 10-P9.larva, 11-P9.larva, 13-P9.larva^d^, 14-P9.larva, 15-P9.larva A6-P20.adult (II), B6-P20.adult (II), C6-P20.adult (II), D6-P20.adult (II)	10 (7.35)
LC772974 (cluster 5)	6 Khorramshahr	A6-P6.larva (II), C6-P6.larva (II), D6-P6.larva (II)	3 (2.2)
LC772975 (cluster 1, Iran-spec.)	**7** Khorramshahr	A7-P7.adult (II), C7-P7.larva (II), E7-P7.larva (II), F7-P7.larva (II)	4 (2.94)
LC772976 (cluster 1, Iran-spec.)	**7** Khorramshahr	B7-P7.adult (II)	1 (0.74)
LC772977 (cluster 4)	**18** Ahvaz	E4-P18.larva	1 (0.74)

^a^GenBank accession number; the cluster (cf. Fig. 2) is given is parentheses^b^Patients with *S. stercoralis* worms with different *cox-1* haplotypes are in bold. Note that only patient 13 had worms with *cox-1* haplotypes from different clusters^c^The codes are composed as follows: [coordinates on the sequencing plate]—[patient code]. [developmental stage] ([haplotype at the nuclear HVR-I if known]), het: heterozygous for haplotypes I and II; het II/III: heterozygous for haplotypes II and III

^d^For these worms the *SSU* HVR-IV was confirmed to be haplotype A. [Worm XY] indicates that this worm was used for whole-genome analysis with this name (cf. Figs. 3, 4, 5). For a sortable table with all the information for each worm, see Additional file 3

### *SSU* Haplotypes

Overall, we identified *SSU* HVR-I haplotypes I, II, and III [14, 17, 32], and we mapped the *SSU* haplotypes onto the *cox-1* tree. Haplotypes I and II were present in both major parts of the tree, with haplotype II being the most frequent haplotype, observed in 64 isolates. Haplotype III was only found in all 10 worms (isolated from two patients) of cluster 4 of which the *SSU* HVR-I sequence was determined. Our results showed that haplotype II was distributed over the entire *cox-1* phylogeny. In contrast to earlier reports from Southeast Asia [14, 17], which had reported no or very few heterozygous worms, we found 27 heterozygous individuals (13 adults and 14 larvae) among our samples (Table 2). Interestingly, 18 of them fell into the Iran-specific *cox-1* cluster (Fig. 2, Additional file 1: Fig. S1). In order to gain further insight into the nature of this Iran-specific cluster, we performed whole-genome sequencing on selected individuals (see below).

For the 24 worms, we sequenced the whole genome, plus for an additional seven *S. stercoralis* from seven patients we also determined the *SSU* HVR-IV sequence (for two of these worms the *cox-1* sequence could not be determined, so they are not in Table 2 and Additional file 3). They all showed haplotype A (cf. 14). This haplotype has been previously reported from humans, dogs, and chimpanzees [14, 16, 32]. Three worms (worms 26, 27, 41) showed a mixed signal, with roughly half of the reads being G and the other half A at position 210 according to Barratt et al. [32], which corresponds to position 1454 in AF279916.

### Whole-genome comparison

**Fig. 3:**
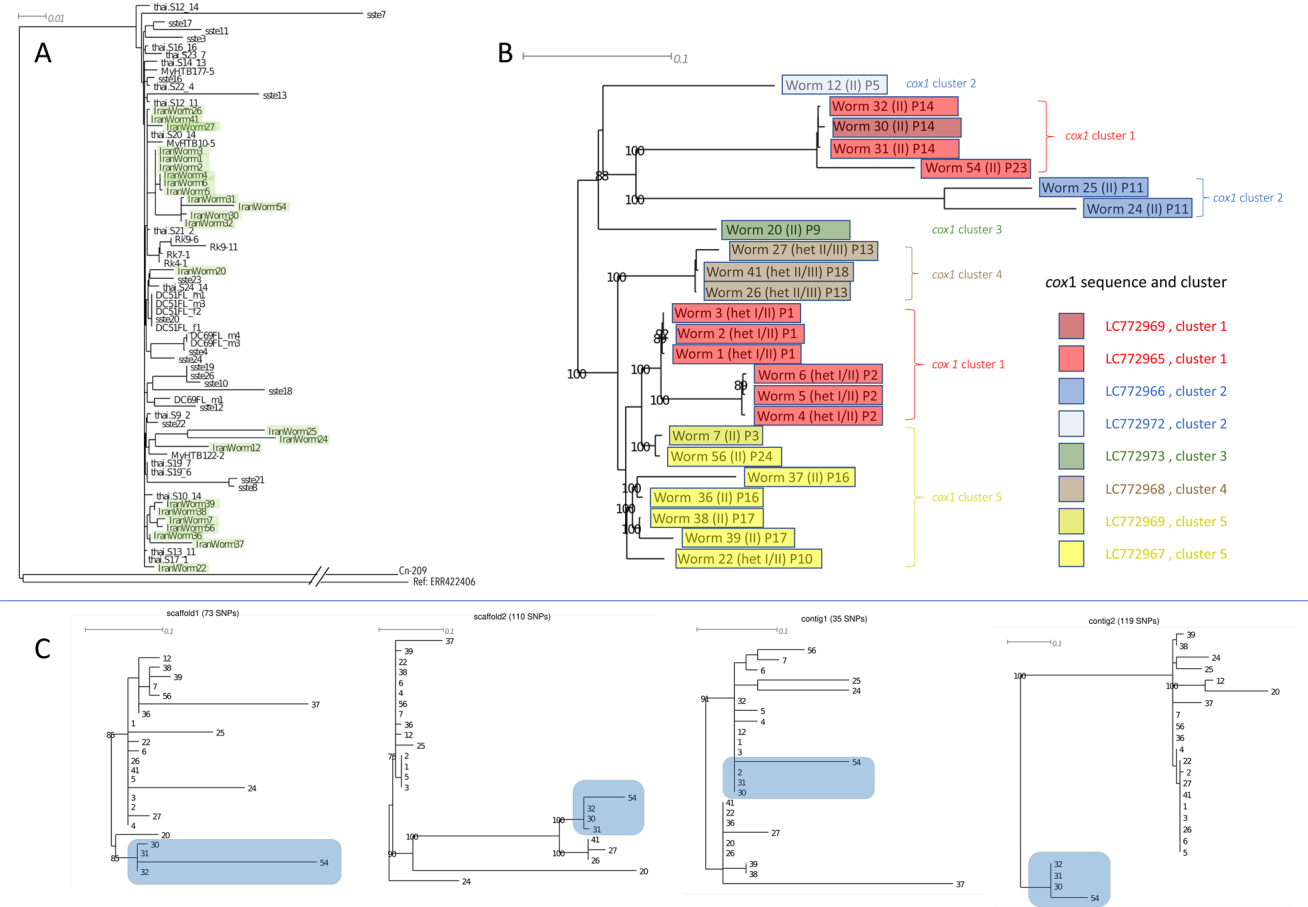
Whole-genome neighbor-joining trees of *Strongyloides stercoralis*. **A** Comparison of the Iranian samples with published sequences from Asia. Cn-209 is an individual from a possibly asexual population in Southern China [19], ERR422406 is one individual of the USA-derived reference isolate [35]. Samples starting with Rk are from Japan [36], new Iranian samples are highlighted in green, and all other samples are from Southeast Asia and are the same as in Fig. 2 of [16]. **B** Iranian samples only. The corresponding *cox-1* haplotypes are color-coded. The *SSU* HVR-I haplotype is indicated. PX: patient [number]. **C** Neighbor-joining trees based on the four largest contigs (genome assembly from Hunt et al. [35]). The resolution is very limited, but examples (one highlighted in blue) with different topology are visible, indicating at least occasional meiotic recombination

**Fig. 4:**
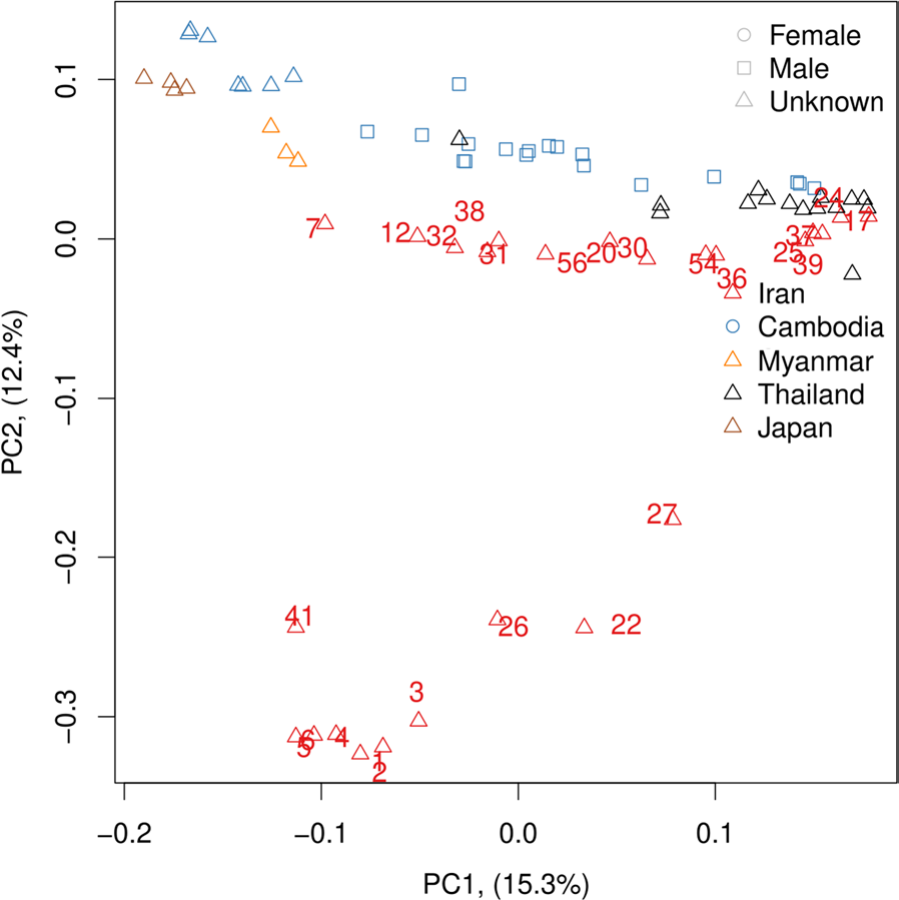
Principal component analysis of whole-genome sequences from Iran and published sequences from Asia. The geographical origin is color-coded. The numbers with the Iranian numbers are the worm numbers (cf. Additional file 1: Fig. 1 and Figs. 2 and 3). The samples from Myanmar and Japan are from [36], the samples from Thailand are from [16], and the samples from Cambodia are from [14]. Note that the sequences from [19] (from Southern China) and the sequences from [35] (from the USA) were not included because they are so different that they dominated the PCA such that no other differences were visible in PC1 and PC2

For 24 worms we managed to sequence the entire genome with coverage above the threshold specified in the Methods section. They were isolated from 14 different patients and represented all the major *cox-1* clusters (Fig. 2, Additional file 1: Fig. S1). In a neighbor-joining cladogram, all sequences fell into the Southeast Asian cluster when compared with the findings of Zhou et. al. [19] (cf. Fig. 4 A of this reference) or Aupalee et al. [16] (cf. Fig. 2 of this reference) (Fig. 3A). However, the resolution within this cluster is low because of the rather small number of variable positions that could be genotyped in all samples (1034 SNPs). Figure 3B shows an unrooted tree with only the sequences from this study. The *SSU* HVR-I haplotypes, the *cox-1* haplotypes and clusters, and the patient of origin are mapped onto the tree. The worms in the Iran-specific *cox-1* cluster appear to fall into two separable groups with respect to their nuclear genome. One group contains the worms with the *SSU* HVR-I haplotype II and the other group the ones that are heterozygous at this locus. Worms from the same patient tend to have very high genomic similarity, both in the bi-parentally inherited nuclear (whole) genome, which can undergo changes through recombination, and in the only maternally inherited, non-recombining, mitochondrial genome. This indicates that they might have shared common ancestors within the last few generations. Possible cryptic species diversity is an issue in parasitic nematodes in general [33] and in *S. stercoralis* in particular [14, 17, 22, 34]. In an attempt to look for recent recombination, we reconstructed trees based on the four largest genomic contigs [35] separately (Fig. 3C). Although the resolution power of these trees is very limited due to the small number of markers, it appears that the four trees differ from each other, indicating that the relationship of the worms differs across the genome. This indicates recent meiotic recombination and suggests that the worms we studied belong to the same and not to multiple cryptic species.

Next, we performed a principal component analysis (PCA) with our sequences and selected published sequences. In this analysis, the Iranian samples also fell within the range of the ones from Southeast Asia and were clearly separated from the sequences reported from South China by Zhou et al. [19] and the reference isolate from the USA [35]. Since these sequences dominated this analysis, we repeated the PCA, excluding them (Fig. 4). Also, in this analysis, the majority of the Iranian samples were very close to those from Southeast Asia, although slightly separated in PC2. However, the samples with a *cox-1* haplotype in the Iran-specific cluster and haplotypes I and II heterozygous *SSU* HVR-I (worms 1, 2, 3, 4, 5, 6) formed a group clearly separated from the rest; in addition, the other haplotype I and II heterozygous *SSU* HVR-I heterozygous worm (worm 22) as well as all three haplotypes II and III heterozygous worms (worms 26, 27, 41) were well separated from the rest in PC2.

**Fig. 5:**
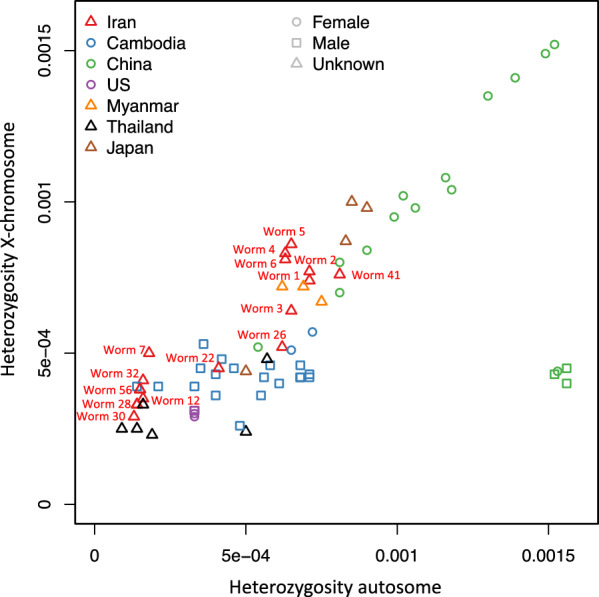
Heterozygosity plot of whole-genome sequences from Iran and published sequences from Asia and the USA (reference isolate [35])). The samples from Myanmar and Japan are from [36], the samples from Thailand are from [16], the samples from China are from [19], and the samples from Cambodia are from [14]. The *X*-axis shows the heterozygosity on the autosomes, the *Y*-axis shows the heterozygosity on the X chromosome. Note that males have only one X chromosome and cannot be heterozygous on this chromosome. The X-chromosomal heterozygosity measured for males is therefore a measure for the error in this measure caused for example by genome assembly and annotation errors

Finally, we analyzed the heterozygosity in our samples (Fig. 5). The Iranian samples fell within the range of the ones from Southeast Asia here as well. Note that many of the samples included for comparison were males, which have only one X chromosome. Therefore, only the autosomal heterozygosity should be compared. However, the Iranian samples formed two distinct groups on either end of the range in Southeast Asia with the worms that were separate from the others in the PCA (Fig. 4) and were heterozygous at the *SSU* HVR-I, showing higher heterozygosity (note that these two analyses are not independent, but heterozygosity may have been a factor in the PCA).

## Conclusions

From all these results we conclude that the *S. stercoralis* population from the Khuzestan province shares much of the genetic diversity with the population in Southeast Asia. However, the presence of an Iran-specific *cox-1* cluster (representing a matrilineage) and the high heterozygosity in the nuclear genome of some individuals indicate a contribution from an additional genetic source. There appears to be some population structure with different subpopulations, which however do interbreed at least occasionally.

### Supplementary Information

**Additional file 1:** cox-1 neighbor-joining tree with all worms from this study listed separately. For comparison, published sequences were included. The tree was constructed using MEGA 7 with the neighbor-joining method and evaluated with 1000 bootstrap repetitions. The evolutionary distances were computed using the Kimura 2-parameter method (using different models resulted in essentially the same tree topology). Scale bar denotes 0.01 changes per nucleotide site. Nomenclature: [worm identifier]-P [patient number].[developmental stage] ([nuclear *SSU* HVR-I haplotype according to [14, 32]]). het: heterozygous for haplotypes I and II, het II/III: heterozygous for haplotypes II and III. Clusters (cf. Fig. 2) are indicated in blue. The worms selected for whole-genome sequencing are indicated in red. Note that not all whole-genome sequencing fulfilled the inclusion quality criteria for all analyses. Therefore, not all the indicated worms are included in Figs. 3, 4 and 5. *These sequences from [14] were found in humans and in dogs and are therefore listed twice. ^+^Note that *SSU* HVR-I haplotypes II and III differ only by one nucleotide (TTT in haplotype II and TAT in haplotype III). Distinguishing homozygous III and heterozygous II/III is therefore not obvious. All three whole-genome-sequenced worms of this group turned out to be heterozygous although one of them had been scored as homozygous for III based on the HVR-I sequencing alone.

**Additional file 2:** Estimates of evolutionary divergence between the different *cox-1* sequences.

**Additional file 3:** Sortable Excel table with all available information for each worm. For nomenclature see legends to Table 2.

## Data Availability

All data supporting the findings of this study are available within the article and/or its supplementary materials or were deposited in publicly available databases.

## Abbreviations

NTD: Neglected tropical disease
HVR: Hypervariable region
*cox-1*: Subunit 1 of mitochondrial cytochrome *c* oxidase gene
APC: Agar plate culture
PCR–RFLP: Polymerase chain reaction–restriction fragment length polymorphism
