# Supplementary figures and images for: Strongyloides stercoralis genotyping in a human population in southwestern Iran
Source: Parasit Vectors. 2024 Jan 16;17:21. doi: 10.1186/s13071-023-06103-6 (PMC10792921; doi:10.1186/s13071-023-06103-6)

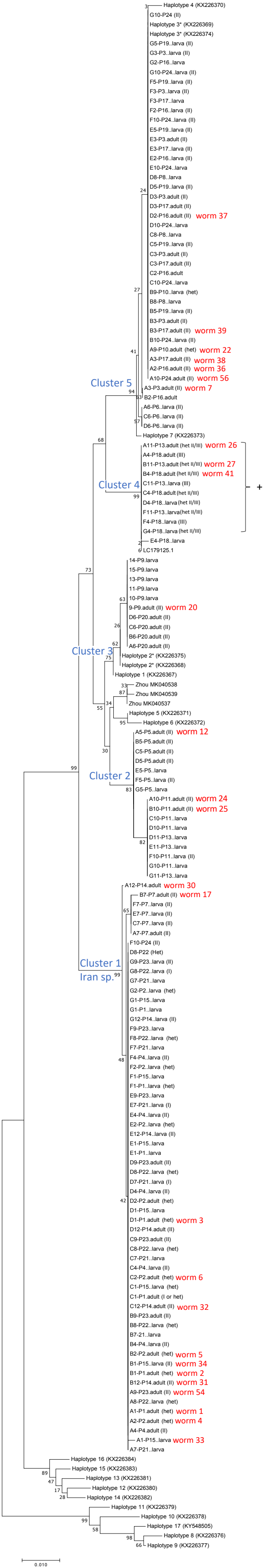

Supplement: Supplementary file 1 — Additional file 1: cox-1 neighbor-joining tree with all worms from this study listed separately. For comparison, published sequences were included. The tree was constructed using MEGA 7 with the neighbor-joining method and evaluated with 1000 bootstrap repetitions. The evolutionary distances were computed using the Kimura 2-parameter method (using different models resulted in essentially the same tree topology). Scale bar denotes 0.01 changes per nucleotide site. Nomenclature: [worm identifier]-P [patient number].[developmental stage] ([nuclear SSU HVR-I haplotype according to [14, 32]]). het: heterozygous for haplotypes I and II, het II/III: heterozygous for haplotypes II and III. Clusters (cf. Fig. 2) are indicated in blue. The worms selected for whole-genome sequencing are indicated in red. Note that not all whole-genome sequencing fulfilled the inclusion quality criteria for all analyses. Therefore, not all the indicated worms are included in Figs. 3, 4 and 5. *These sequences from [14] were found in humans and in dogs and are therefore listed twice. +Note that SSU HVR-I haplotypes II and III differ only by one nucleotide (TTT in haplotype II and TAT in haplotype III). Distinguishing homozygous III and heterozygous II/III is therefore not obvious. All three whole-genome-sequenced worms of this group turned out to be heterozygous although one of them had been scored as homozygous for III based on the HVR-I sequencing alone. [file 13071_2023_6103_MOESM1_ESM.pdf]
